# Quantitative Real-Time PCR Assays for the Detection of Pathogenic *Leptospira* Species in Urine and Blood Samples in Canine Vaccine Clinical Studies: a Rapid Alternative to Classical Culture Methods

**DOI:** 10.1128/JCM.03006-20

**Published:** 2021-06-18

**Authors:** Stephanie Blanchard, Carine Cariou, Jérôme Bouvet, William Valfort, Frantz Oberli, Séverine Villard, Florence Barret-Hilaire, Hervé Poulet, Lionel Cupillard, Blandine de Saint-Vis

**Affiliations:** a Boehringer Ingelheim Animal Health, Saint-Priest, France; b Boehringer Ingelheim Animal Health, Saint-Vulbas, France; University of Tennessee at Knoxville

**Keywords:** pathogenic *Leptospira* spp., clinical study, *lipL32* gene, real-time PCR

## Abstract

Leptospirosis is a vaccine-preventable bacterial zoonotic disease caused by pathogenic Leptospira species. The efficacy of *Leptospira* canine vaccines is assessed by challenging vaccinated and control dogs with virulent serovars of *Leptospira*, followed by detection of *Leptospira* in blood and urine. We assessed the consistency between results obtained for urine and blood samples from clinical studies with species-specific real-time quantitative PCR (qPCR) targeting the *lipL32* gene and those obtained with the reference culture method. The specificity of the qPCR assay was confirmed by negative results for nonpathogenic *Leptospira* and for several canine viruses, bacteria, and parasites. The results from the two methods were compared using McNemar’s test, kappa coefficient (κ), and percentage of agreement analyses. The results for numbers of positive and negative dogs were similar, with no false-negative results with the qPCR assay. For both blood and urine, there was strong agreement between the culture method and qPCR results (κ = 0.68 [95% confidence interval (CI), 0.62 to 0.74] and κ = 0.65 [95% CI, 0.59 to 0.71], respectively). However, there was a statistically significant difference between blood samples (*P* < 0.001) and urine samples (*P* = 0.028). The negative percentage agreements were 97% and 84% and the positive percentage agreements were 68% and 83% for blood and urine samples, respectively. Although the cell culture method is the recommended gold standard, our results show that qPCR assay is a valid alternative method for the rapid and specific detection of pathogenic *Leptospira* spp. in urine and blood samples during vaccine efficacy studies, without loss of sensitivity.

## INTRODUCTION

Canine leptospirosis is an infection found in both domestic and wild dogs that is caused by pathogenic Gram-negative spirochaete bacteria belonging to the genus Leptospira. Clinical signs associated with *Leptospira* infection in dogs range from subclinical to severe and potentially fatal disease with multiorgan dysfunction (1–4). Leptospirosis is one of the most widespread zoonotic diseases and infects both human and animals. Infection occurs through direct contact with infected animal urine or indirectly through water or soil contaminated with infected urine from rodents and other wild animals (1). Vaccination is recommended in dogs that are potentially exposed to pathogenic *Leptospira* strains to protect them from the disease and to reduce the zoonotic risk by reducing shedding of virulent *Leptospira*.

LipL32 (also known as Hap1), an outer membrane, surface-exposed lipoprotein, is the most abundant leptospirosis protein that is found exclusively in pathogenic leptospires. This highly conserved protein among pathogenic *Leptospira* strains is expressed *in vivo* and is very immunogenic. Antibodies against LipL32 are detected during all stages of the disease and have been shown to be highly specific, with no detectable cross-reactivity to unrelated antigens (5–7).

During clinical development, the efficacy of veterinary *c* vaccines is assessed by their ability to protect dogs against challenge with specific pathogenic *Leptospira* serovars (8). The European Pharmacopoeia requires vaccine efficacy to be assessed by evaluating the onset of immunity (OOI) and the duration of immunity (DOI), using protection against challenge with a pathogenic strain. Protection is assessed using clinical parameters and by testing blood, urine, and kidney tissue samples from challenged dogs for the presence of *Leptospira* using a reference culture method for up to 6 weeks after challenge. This reference culture method is laborious and time-consuming, since *Leptospira* growth is slow and can take several weeks, and the cultures must be checked for live leptospires under a light microscope at regular intervals. This lengthy multistep process for culture and reading increases the risk of contamination of the test samples and the potential risk of exposure for technicians.

Real-time quantitative PCR (qPCR) is a molecular biology technique for *in vitro* amplification of nucleic acids. Assays based on qPCR have been developed for leptospirosis diagnosis and have been shown to be faster, easier, and less labor intensive than the culture method, with acceptable sensitivity and specificity for routine diagnosis in whole blood and serum (9, 10).

The potential advantages of using qPCR assays for vaccine clinical studies are similar and include being able to obtain the results faster than with the reference cell culture methods. In addition, samples can be stored frozen, enabling extraction to be performed later and repeated if necessary, unlike samples for the cell culture method that must be assayed extemporaneously after reception. They are also less labor intensive and require less handling of potentially pathogenic species in the laboratory, thereby reducing the risk of infection for technicians. To be considered valid tests for regulatory requirements, their sensitivity must be at least equivalent to that of the reference cell culture methods.

The most sensitive and specific qPCR assays for the detection of pathogenic *Leptospira* species target the *LipL32* gene (9). We evaluated a LipL32 qPCR assay using samples from *Leptospira* vaccine clinical efficacy studies and compared the results with those obtained with the reference culture method (gold standard). The aim of this evaluation was to compare the results for the blood and urine samples and to verify if the conclusions about vaccine efficacy were consistent in the studies with both methods.

## MATERIALS AND METHODS

### Vaccines.

The following vaccines were used in the clinical studies, as indicated in Table 1.

**TABLE 1:** Summary of study characteristics

Study no.	Injection no.	Vaccine	Dose	Challenge strain	No. of dogs	
					Vaccinated	Control
Onset-of-immunity studies						
Study 1	1	L3				
	2	L3	1/4	Li	6	6
Study 2	1	Versican DHPPi-L3				
	2	Versican DHPPi-L3R	Full	Li	6	6^*a*^
Study 3	1	DAPPi-L3				
	2	DAPPi-L3 + Rabisin	Full	Lc	6	6
Study 4	1	L3				
	2	L3	1/4	Lc	6	6^*b*^
Study 5	1	L3				
	2	L3	1/4	Lg	6	6
Study 6	1	Versican DHPPi-L3				
	2	Versican DHPPi-L3R	Full	Lg	6	6^*c*^
Duration-of-immunity studies						
Study 7	1	DAPPi-L3				
	2	DAPPi-L3 + Rabisin	Full	Lg	7	6
Study 8	1	DAPPi-L3				
	2	DAPPi-L3 + Rabisin	Full	Li	7	6

a Same animals as in study 1.

b Same animals as in study 3.

c Same animals as in study 5.

• Eurican DAPPi-Lmulti (Boehringer Ingelheim), a freeze-dried vaccine containing four live attenuated viruses, namely canine distemper virus, canine adenovirus type 2, canine parvovirus type 2, and canine parainfluenza virus (Eurican DAPPi), combined with a liquid vaccine containing three inactivated strains of Leptospira interrogans (serovars Canicola, Icterohaemorrahgiae, and Grippotyphosa) (Eurican L multi).
• L3 (Boehringer Ingelheim; batch 2485D5S401), equivalent to the commercialized vaccine Eurican L multi.
• DAPPi (Boehringer Ingelheim; batch 2485P5D272), equivalent to the commercialized vaccine Eurican DAPPi.
• Rabisin (Boehringer Ingelheim; batch 1RBNS0151), an inactivated, adjuvanted vaccine containing rabies glycoproteins.
• Versican DHPPi/L3 (Zoetis; batch 15601901), containing the same valences as Eurican DAPPi-Lmulti.
• Versican DHPPi/L3R (Zoetis; batch 19581901), containing the same valences as Eurican DAPPi-Lmulti, as well as inactivated rabies.

### Clinical study samples.

**FIG 1:**
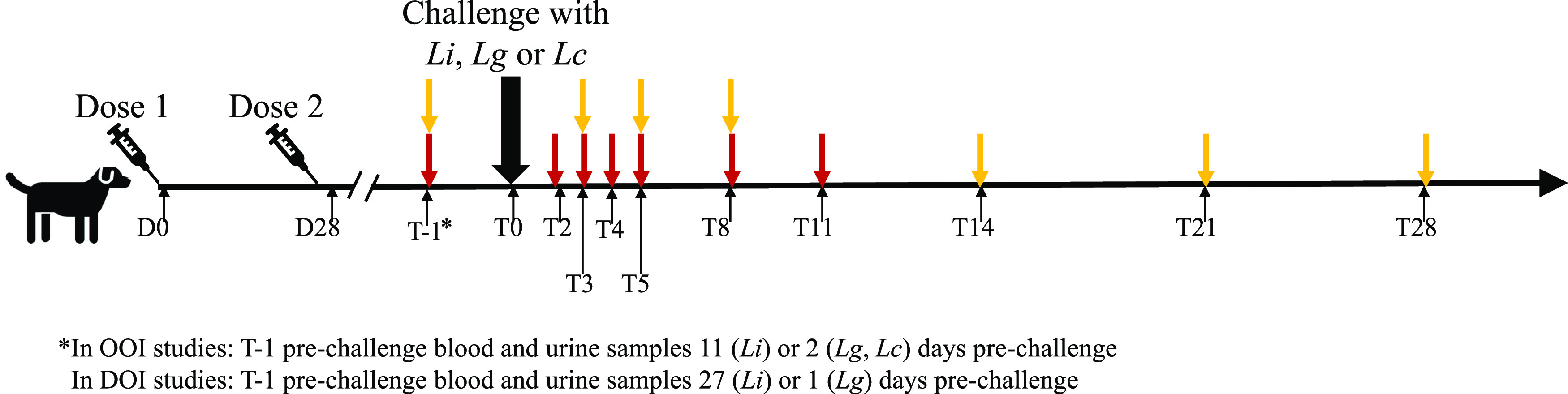
Schematic representation of the onset of immunity and duration of immunity clinical study design. Time point zero (T0) was 2 weeks after the second injection of L3 or 3 weeks after Veriscan vaccination for the onset-of-immunity (OOI) studies and about 1 year for the duration-of-immunity (DOI) studies. Red arrows represent time points for blood samples, and yellow arrows represent time points for urine samples. Lc, Leptospira interrogans serovar Canicola; Lg, Leptospira interrogans serovar Grippotyphosa; Li, Leptospira interrogans serovar Icterohaemorrhagiae.

Samples from six OOI and two DOI clinical studies were tested for the presence of *Leptospira* using the qPCR assay (Table 1). In these clinical studies, the vaccinated puppies received subcutaneous injections of vaccines containing Leptospira interrogans serovars (*L.* Canicola, *L.* Icterohaemorrhagiae, and *L.* Grippotyphosa) on days 0 and 28 (Table 1 and Fig. 1). The control puppies were either unvaccinated or had received Rabisin, an adjuvanted, inactivated rabies vaccine (Boehringer Ingelheim). Two weeks after the second injection (or 3 weeks after Versican vaccination) in the OOI studies and at about 1 year in the DOI studies, vaccinated and control puppies were challenged with specific virulent serovar suspensions. The blood and urine samples were collected at defined time points and assessed for the presence of *Leptospira* spp. using the classical culture method as described previously (8, 11, 12). The dogs were considered to be positive and to have leptospiremia or leptospiruria if at least one blood or urine culture, respectively, was positive at any time point.

In the OOI studies, blood samples were taken 2 days (for *L*. Canicola and *L.* Grippotyphosa challenge studies) or 11 days (for *L.* Icterohaemorrhagiae challenge studies) prechallenge, and at 2, 3, 4, 5, 8, and 11 days postchallenge, and urine samples were taken 2 days (for *L*. Canicola and *L.* Grippotyphosa challenge studies) or 11 days (for *L.* Icterohaemorrhagiae challenge studies) prechallenge, and at 3, 5, 8, 10, 14, 21, and 28 days postchallenge (Fig. 1). In the DOI studies, prechallenge blood and urine samples were taken at 1 and 27 days before *L.* Grippotyphosa and *L.* Icterohaemorrhagiae challenges, respectively, and the postchallenge samples were taken at the same times as in the OOI studies. Thus, there were seven time points for blood samples and eight time points for urine samples for each dog, if the dogs survived until the end of the study. The EDTA blood samples were stored frozen at between −20°C and −70°C and urine samples were stored at +5°C for up 1 week before DNA extraction.

All dogs were ethically euthanized, either when a predefined clinical outcome occurred or at the end of the study. The protocols for all clinical studies were reviewed by the Boehringer Ingelheim’s Ethics Committee (registered under number 13 at the French Ministère de l’Education Nationale, de l’Enseignement Supérieur et de la Recherche) before conducting the studies. Approval confirmed that all experiments conformed to the relevant regulatory standards defined by the European and French Laws (directive EU2010/63 and Decret no. 2013-118).

### Sample collection.

Urine was collected after subcutaneous injection of furosemide (2.5 to 5 mg/kg bodyweight) via urinary catheterization, cystocentesis, or direct bladder tap at the time of necropsy. The urine samples were either used immediately or stored at +5°C for up to 7 days before use. Blood samples were collected and either used immediately or frozen at −20°C for up to 15 days before use.

### DNA extraction from blood and urine samples.

DNA was extracted from 100 μl of the thawed EDTA blood samples, diluted in 100 μl of 1× phosphate-buffered saline (PBS) buffer, using a QIAamp DNA blood minikit (Qiagen, Valencia, CA), with elution into 200 μl of buffer AE (10 mM Tris-Cl and 0.5 mM EDTA [pH 9.0] supplied by the manufacturer).

Prior to DNA extraction from the urine samples, 1 ml was centrifuged at 2,000 × *g* for 10 min and the pellet suspended in 200 μl of lysis buffer. The DNA was then extracted using the QIAamp DNA blood minikit with elution into 50 μl of buffer AE.

### Primers and probes for the qPCR assays.

The qPCR assays were designed to amplify the *LipL32* gene, which is specific to pathogenic *Leptospira* strains, in samples obtained from dogs following challenge with virulent *Leptospira* sp. in the setting of clinical trials assessing vaccine efficacy. Sequences of the *LipL32* gene from 17 L. interrogans serovars were aligned using vector NTI software (Thermo Fisher Scientific) (Fig. 1). Previously described primer and probe sequences were tested and optimized *in silico* using Beacon Designer software (Premier Biosoft) (see Fig. S1 in the supplemental material) (9). The optimization required a slight modification of the sequence for the forward primer (Fig. 1). The forward *LipL32*-F (5′-CTG TGA TCA ACT ATT ACG GAT A-3′) and reverse *LipL32*-R (5′-GAA CTC CCA TTT CAG CGA T-3′) primers were used to amplify a 123-bp fragment of the *LipL32* gene. The amplification was detected by a *LipL32*-P TaqMan probe (6-carboxyfluorescein [FAM]-5′-AAA GCC AGG ACA AGC GCC G-3′-black hole quencher 1 [BHQ1]). The specificity of the primers was evaluated using DNA extracted from pathogenic and nonpathogenic *Leptospira* strains and canine viruses.

### Internal control.

To assess the presence of inhibitors that could result in false-negative PCR results, we spiked the blood and urine samples after lysis but before DNA extraction with a plasmid containing a sequence coding for green fluorescent protein (GFP) as an internal control (4.10^5^ copies/5 μl for blood samples and 5 × 10^4^ copies/5 μl for urine samples). This control DNA was amplified at the same time as the *LipL32* gene during the qPCR assay using forward and reverse primers (GFP-F and GFP-R) and a GFP TaqMan probe (Eurofins, France).

### Standard plasmid.

A DNA construct was manufactured to evaluate the sensitivity of the qPCR and to calculate the amplification efficiency using the formula 10^−1/slope^ − 1 for the standard curve (13). This construct was used to amplify 302 bp of the *lipl32* sequence from L. interrogans serovar Canicola by conventional PCR, using a *Taq* PCR master mix kit (Qiagen). The PCR product was ligated into a pCR4-Topo plasmid (Thermo Fisher Scientific), transformed into One Shot DH5α bacteria (Thermo Fisher Scientific), and then selected using blue/white screening according to the manufacturers’ recommendations. Plasmid DNA was extracted from positive clones using the plasmid midikit (Qiagen), DNA concentration was determined from the measure of optical density at 260 nm (OD_260_), and purity was evaluated by calculating the OD_260_/OD_280_ ratio. The number of copies of plasmid was calculated, and the plasmid concentration was standardized to 1E+08 copies/5 μl with Tris-EDTA buffer (pH 8).

### Optimization of real-time PCR assay conditions.

Different primers and probes concentrations, annealing temperatures, and incubation times were assessed to determine the optimal conditions for the duplex real-time PCR assay. The qPCR amplification was performed in a CFX96 Touch system (Bio-Rad), using QuantiTect multiplex PCR master mix (Qiagen), in a total volume of 25 μl containing 12.5 μl of 2× ReadyMix reagent, 200 nM *lipL32* primers and probe, 100 nM GFP primers and probe, and 5 μl sample DNA. The qPCR amplification program required incubation for 10 min at 95°C to activate the *Taq* polymerase, followed by 39 cycles at 95°C for 30 s to denature the nucleic acid, followed by 1 cycle at 1 min at 60°C for hybridization and elongation. Nuclease-free water replaced the extracted DNA in the negative control. Duplicate samples and negative controls were analyzed for reproducibility. Tenfold dilutions of the plasmid construct in Tris-EDTA buffer (pH 8) containing from 1E+06 to 1E+01 copies number per reaction were included in all assays. The results for these dilutions were used to obtain a standard curve and to determine the amplification efficiency.

### Statistical analyses.

The objective of the statistical analysis was to assess the agreement of the results from the reference culture method and the qPCR assay. If at least one culture or qPCR result was positive, the sample was considered to be positive. If discordant results were obtained for the duplicates in the qPCR assay (i.e., one positive and one negative) the sample was considered positive.

McNemar’s test was used to evaluate if there were systematic differences between the culture and the qPCR results (14, 15). A *P* value of ≤0.05 indicated that the results are dependent on the method, i.e., a systematic difference exists. A *P* value of >0.05 indicated that there is no evidence of a systematic effect, but this was not evidence that there was agreement. Cohen’s kappa coefficient (κ) was used to measure the difference between the observed agreement and the agreement that could be expected due to chance alone (16). The values for the κ coefficient range from −1 to 1, with 1 indicating complete agreement, −1 complete disagreement, and 0 agreement that could be obtained by chance alone. The level of agreement was assessed using the scale of the κ coefficient (17). A κ value of <0 indicated no agreement; 0 to 0.20 indicated very weak agreement, 0.21 to 0.40 indicated weak agreement, 0.41 to 0.60 indicated moderate agreement, 0.61 to 0.80 indicated strong agreement, and 0.81 to 1.00 indicated almost complete agreement.

All statistical analyses were performed with SAS 9.4 commercial software (Cary, NC). Hypothesis tests and point estimates were two-tailed, with a significance threshold set at an α of 5%.

### Data availability.

Data will be made available upon reasonable request to the corresponding author.

## RESULTS

### Specificity of qPCR method for the detection of *Leptospira*.

Nucleic acid from all *Leptospira* spp. tested was amplified in the qPCR assay, apart from Leptospira biflexa Patoc, which is a nonpathogenic strain (see Table S1 in the supplemental material). Nucleic acid from the canine viruses or from nonpathogenic *Leptospira* species listed in the table was not amplified, confirming the specificity of the probes for pathogenic *Leptospira*. Amplification of the internal control nucleic acid (GFP) was observed in all qPCRs, including the negative control, indicating no inhibition of the PCRs from the various components in the samples. The limit of detection of the qPCR was below 10 copies/reaction in both blood and urine (internal data, not shown).

The efficiency of the qPCR was evaluated using the standard curve constructed with the results for reference samples containing 10 to 10^7^ copies/ml of the *Lipl32* plasmid. We observed a linear relationship between the starting copy number and the threshold cycle, with an *R*^2^ of 0.999 and an efficiency (E) of 100%.

### Analyses of clinical study samples.

**FIG 2:**
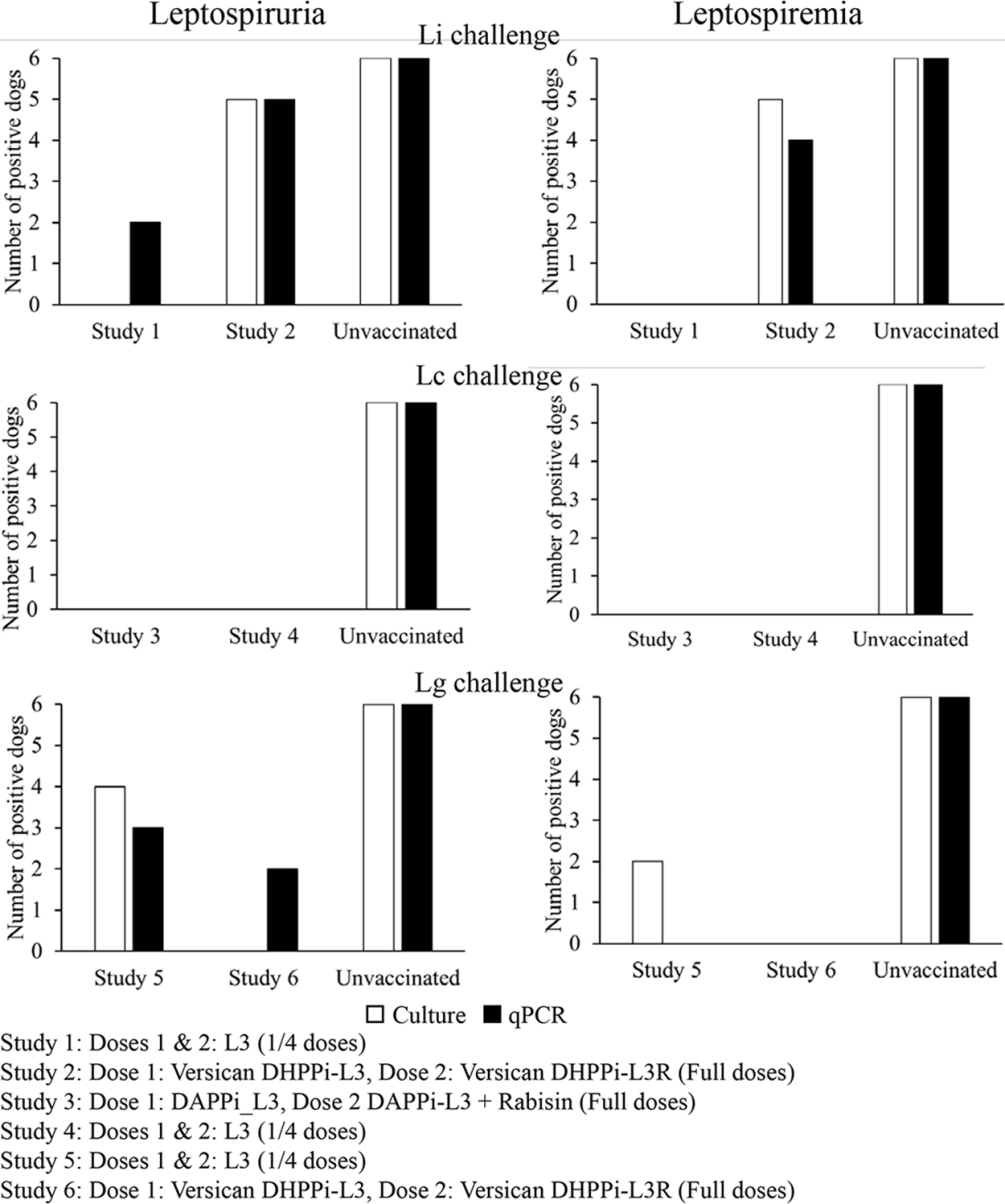
Results from six onset of immunity studies in which dogs were challenged with *L.* Canicola (Lc), *L.* Grippotyphosa (Lg), or *L.* Icterohaemorrhagiae (Li), as indicated. Number of positive dogs per study group with leptospirae in blood and urine samples detected using culture method (white bars) and quantitative PCR (qPCR) assay (black bars).

The results for blood and urine samples from the six OOI studies in which *Leptospira* serovars Canicola, Grippotyphosa, or Icterohaemorrhagiae were used as the challenge are summarized in Fig. 2.

The detailed results from study 3 are shown as an example to illustrate the consistent interpretation of the culture and qPCR results with both methods (Table 2). All six dogs in the vaccinated group were negative by both the culture method and qPCR, corresponding to complete protection. All six dogs in the control group were positive by both methods for at least one time point, indicating no protection.

**TABLE 2:** Example of detailed results for blood samples taken from vaccinated (A and C) and control dogs (B and D)^*a*^

Method, dog vaccination status, and dog no.	Time point							No. of dogs positive
	Prechallenge	T2	T3	T4	T5	T8	T11	
Reference culture								
A. Vaccinated								
1	−/−	−/−	−/−	−/−	−/−	−/−	−/−	
2	−/−	−/−	−/−	−/−	−/−	−/−	−/−	
3	−/−	−/−	−/−	−/−	−/−	−/−	−/−	
4	−/−	−/−	−/−	−/−	−/−	−/−	−/−	
5	−/−	−/−	−/−	−/−	−/−	−/−	−/−	
6	−/−	−/−	−/−	−/−	−/−	−/−	−/−	0
B. Control								
7	−/−	+/+	+/+	+/+	+/+	—^*b*^	—^*b*^	
8	−/−	+/+	+/+	+/+	+/+	+/+	+/+	
9	−/−	+/+	+/+	+/+	+/+	−/−	−/−	
10	−/−	+/+	+/+	+/+	+/+	+/+	−/−	
11	−/−	+/+	+/+	+/+	+/+	+/+	−/−	
12	−/−	+/+	+/+	+/+	+/+	+/+	−/−	6
qPCR								
C. Vaccinated								
1	−/−	−/−	−/−	−/−	−/−	−/−	−/−	
2	−/−	−/−	−/−	−/−	−/−	−/−	−/−	
3	−/−	−/−	−/−	−/−	−/−	−/−	−/−	
4	−/−	−/−	−/−	−/−	−/−	−/−	−/−	
5	−/−	−/−	−/−	−/−	−/−	−/−	−/−	
6	−/−	−/−	−/−	−/−	−/−	−/−	−/−	0
D. Control								
7	−/−	+/+	+/+	+/+	+/+	—^*b*^	—^*b*^	
8	−/−	+/+	+/+	+/+	+/+	+/−	−/−	
9	−/−	+/+	+/+	+/−	−/−	−/−	−/−	
10	−/−	−/−	+/−	+/+	+/+	+/+	−/−	
11	−/−	+/+	+/+	+/−	+/+	−/−	−/−	
12	−/−	−/−	−/−	+/+	+/+	−/−	+/−	6

a With the reference culture method and qPCR assay from study 3 (a challenge study with *L.* Canicola). For qPCR, the result was considered positive if at least one of the duplicates was positive (+/−). The dogs were considered positive if one positive result was observed at any time point. Entries with two positive results are shaded dark gray, and entries with one positive and one negative result are shaded light gray.

b Dog euthanized at time point 5 (T5) after developing severe leptospirosis.

**FIG 3:**
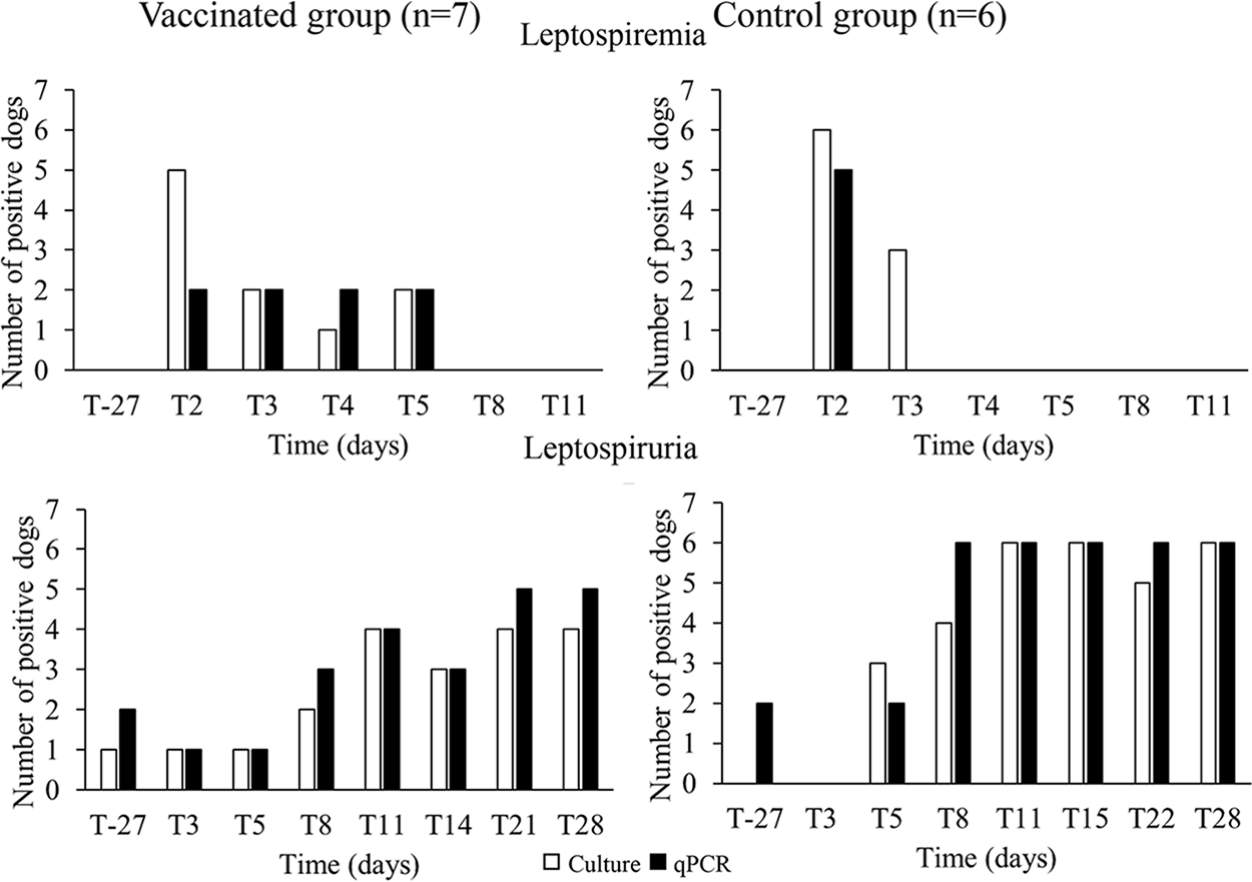
Example of results with culture method (white bars) and qPCR assay (black bars) in blood and urine samples from one of the duration-of-immunity studies (study 7), in which dogs were challenged with *L.* Grippotyphosa (Lg). The numbers of positive dogs at each time point in the vaccinated and control groups are shown.

All dogs in vaccinated and control groups in both DOI studies had at least one positive blood sample and one positive urine sample and were therefore considered positive (infected). The evolution of the number of dogs with positive blood and urine samples over time from study 7, in which the dogs received one dose of DAPPi-L3 and one dose of DAPPi-L3 and Rabisin, is shown as an illustration in Fig. 3.

### Agreement between culture and qPCR methods.

The same results were observed for 475 (85.1%) blood samples and 499 (83.3%) urine samples with both the reference culture method and the qPCR assay (positive or negative) (Table 3). Among the 229 (41.0%) and 228 (38.1%) blood and urine samples, respectively, that were positive by culture, 74 (13.3%) and 39 (6.5%), respectively, were negative by qPCR. Among the 329 (59.0%) and 371 (61.9%) blood and urine samples, respectively, that were negative by culture, 9 (1.6%) and 61 (10.2%), respectively, were positive by qPCR. Thus, the percent negative and positive agreements were 97% and 68%, respectively, for blood samples and 84% and 83%, respectively, for urine samples.

**TABLE 3:** Summary of frequency and percentage of Leptospira detection by culture and qPCR methods^*a*^

Detection parameter	qPCR					
	Blood			Urine		
	Negative	Positive	Total	Negative	Positive	Total
Culture						
Negative	**320 (57.3)**	9 (1.6)	329 (59.0)	**310 (51.7)**	61 (10.2)	371 (61.9)
Positive	74 (13.3)	**155 (27.8)**	229 (41.0)	39 (6.5)	**189 (31.6)**	228 (38.1)
Total	394 (70.6)	164 (29.4)	558 (100.0)	349 (58.3)	250 (41.7)	599 (100.0)
*P* value^*b*^	<0.001			0.028		
κ (95% CI)^*c*^	0.68 (0.62–0.74)			0.65 (0.59–0.71)		
Positive % agreement	68			83		
Negative % agreement	97			84		

a Numbers of samples that gave the same result (positive or negative) are shown in boldface type.

b McNemar’s test for systematic difference.

c Cohen’s kappa coefficient (κ) with 95% confidence interval (CI).

For both blood and urine, there was strong agreement between the reference culture method and the qPCR (κ = 0.68 [95% CI, 0.62 to 0.74] and κ = 0.65 [95% CI, 0.59 to 0.71], respectively), However, there were statistically significant systematic differences in blood samples (*P* < 0.001) and in urine samples (*P* = 0.028).

## DISCUSSION

During clinical development, the efficacy of veterinary *Leptospira* vaccines to protect dogs against challenge with specific virulent *Leptospira* serovars is assessed (8). Protection is assessed using clinical criteria and by testing blood, urine, and kidney tissue samples from challenged dogs for the presence of *Leptospira* using a reference culture method for up to 6 weeks after challenge, in accordance with the European Pharmacopoeia. Here, we showed the specificity of the *Lipl32* qPCR for pathogenic *Leptospira* spp. with no cross-reaction with viral or other bacterial pathogens and no false-positive or false-negative signals from blood or urine samples (9). We demonstrated that the results from the *Lipl32* qPCR assay resulted in the same conclusions about the number of dogs with positive and negative blood and urine samples as those from the culture method. Overall, the qPCR assay results for the blood and urine samples from the OOI and DOI studies were consistent with those obtained with the reference cell culture method, with a kappa coefficient that indicated strong agreement between the methods (κ = 0.68 [95% CI, 0.62 to 0.74] and κ = 0.65 [95% CI, 0.59 to 0.71], respectively) (Tables 1 and 3). The results for the *L.* Grippotyphosa and *L.* Icterohaemorrhagiae challenge studies seemed to be less consistent than the results for the *L.* Canicola challenge studies, but this did not change the overall conclusions of the studies.

In the OOI study 3, assessing a full dose of the DAPPi-L3 vaccine (which is now commercially available as the Eurican DAPPi-Lmulti vaccine), we demonstrated that both the reference culture method and the qPCR assay for *L.* Canicola, *L.* Icterohaemorrhagiae, and *L.* Grippotyphosa in blood and in urine showed the absence of *Leptospira* in blood and in urine in the vaccinated dogs, confirming a sterile immunity for all *Leptospira* valences contained in the Eurican DAPPi-Lmulti vaccine (8, 12). In OOI studies 1, 4 and 5, where the dogs received one-quarter of the L3 vaccine dose, some had *Leptospira*-positive urine and blood after challenge because they had been given a lower dose of vaccine. This enabled us to compare the performance of the two methods in samples from dogs with different levels of infection and showed that the conclusions from the two methods were the same. The results for the samples from these studies were similar in both urine and blood for the three challenges, with no positive results in urine from the *L.* Canicola- and *L.* Icterohaemorrhagiae-challenged dogs (Fig. 2). Two of the *L.* Grippotyphosa-challenged dogs had positive urine results with the qPCR assay but not with the cell culture assay, which may be explained by the greater sensitivity of the qPCR assay in urine, since it can detect even traces of LipL32 DNA, unlike the culture method, which requires the presence of live *Leptospira*. In the studies with different competitor vaccines in our severe challenge model, we obtained similar numbers of positive blood and urine samples from vaccinated dogs.

The agreement between the assays was verified by comparing results from both methods for all the samples. Overall, 475 (85.1%) blood samples and 499 (83.3%) urine samples had the same result with both methods. The positive percent agreement was 68% for blood samples compared with 83% for urine samples, largely due to 74 (13.3%) blood samples that were negative by qPCR but positive by the culture method. In contrast, the negative percent agreement was high for both the blood and urine samples—97% and 84%, respectively. These results show that the sensitivity of the qPCR assay is excellent in urine samples, and although it is slightly lower in blood samples, there is no impact on the global results. The limit of detection of *Leptospira* in urine and blood is 7 and 1 copy number/sample (i.e., 7 and 1 leptospire/test), respectively (internal data, not shown) and therefore is appropriate for monitoring samples from vaccine clinical studies. This difference in sensitivity can be explained since urine is a uniform liquid matrix, in contrast to blood, which is a complex, viscous matrix containing cellular material and, potentially, inhibitors, making it a less favorable environment for PCR amplification. In addition, the sampling and pretreatment of the two matrixes were optimized to take into consideration their physiochemical characteristics and the nature of *Leptospira*. The pretreatment for urine samples includes a 10-fold concentration step before lysis, which explains the high sensitivity in urine. As mentioned above, blood is a more complex, viscous matrix and therefore concentration was not possible, but the sample pretreatment was optimized to prevent potential inhibition of the PCR by the diverse components present in blood.

It is important to note that despite these differences, the qPCR assay did not result in more false-negative results, i.e., falsely concluding that dogs in the clinical studies were protected by vaccination. In vaccine efficacy studies, the presence of *Leptospira* in kidneys is also assessed; however, there are fewer samples in each study because the dog is sacrificed to obtain the kidney sample for testing. We therefore did not test kidney samples during our studies, but the qPCR assay is now used in vaccine efficacy studies for urine, blood, and kidney samples (12).

The specificity and sensitivity of qPCR assays for routine diagnosis of human leptospirosis have been demonstrated (9, 10, 18–21). The diagnosis can be confirmed in the early phase of the disease (within 2 weeks of exposure) with these qPCR assays, before detectable levels of antibodies are present.

The reference culture method is laborious and time-consuming, as *Leptospira* growth in culture can take several weeks. Furthermore, it is sensitive to contamination that can mask the presence of *Leptospira*. The advantages of using the qPCR assay in vaccine clinical development include being able to obtain results that are consistent with those obtained from the reference cell culture methods, more rapidly. In addition, samples can be frozen and stored, enabling assays to be performed later and, if necessary, to be repeated, unlike samples for the cell culture method, which must be processed extemporaneously. They are also less labor intense and require less handling of potentially pathogenic species in the laboratory, and hence the risk of infection for the operators and the risk of contamination between samples are reduced.

Canine challenge studies are required by the European Pharmacopeia Monograph 0447 for assessment of inactivated canine leptospirosis vaccines. The registration of the claim of cross-protection against *L. interrogans* serovar Copenhageni in Europe for the Eurican DAPPi-Lmulti vaccine was based on results from this qPCR method (12). In the future, we will be using the qPCR method only to assess vaccine efficacy with the *Leptospira* challenge model, strengthening the interest of the method. Although the cell culture method is considered to be the gold standard, our results show that qPCR assay is a valid alternative method for the rapid and specific detection of pathogenic *Leptospira* spp. in urine and blood samples during vaccine efficacy studies, without loss of sensitivity.
