# Supplementary material for: Quantitative Real-Time PCR Assays for the Detection of Pathogenic Leptospira Species in Urine and Blood Samples in Canine Vaccine Clinical Studies: a Rapid Alternative to Classical Culture Methods
Source: J Clin Microbiol. 2021 Jun 18;59(7):e03006-20. doi: 10.1128/JCM.03006-20 (PMC8218769; doi:10.1128/JCM.03006-20)
Supplement: Supplemental file 1 — Fig. S1. Download JCM.03006-20-s0001.pdf, PDF file, 569 KB [file jcm.03006-20-s0001.pdf]

```
                                        160                                                                         239
L. canicola (AJ580493)            (160) GGATCTGTGATCAACTATTACGGATACGTAAAGCCAGGACAAGCGCCGGACGGTTTAGTCGATGGAAACAAAAAAGCATA
L. canicola (DQ092412)             (97) GGATCTGTGATCAACTATTACGGATACGTAAAGCCAGGACAAGCGCCGGACGGTTTAGTCGATGGAAACAAAAAAGCATA
L. canicola (HM026175)            (160) GGATCTGTGATCAACTATTACGGATACGTAAAGCCAGGACAAGCGCCGGACGGTTTAGTCGATGGAAACAAAAAAGCATA
L.canicola (AY763509)              (58) GGATCTGTGATCAACTATTACGGATATGTAAAGCCAGGACAAGCGCCGGACGGTTTAGTCGACGGAAACAAAAAAGCATA
L. canicola (JN831363)            (160) GGATCTGTGATCAACTATTACGGATACGTAAAGCCAGGACAAGCGCCGGACGGTTTAGCCGATGGAAACAAAAAAGCATA
L. Grippotyphosa (GU592524)       (160) GGATCTGTGATCAACTATTACGGATACGTAAAGCCAGGACAAGCGCCGGACGGTTTAGTCGATGGAAACAAAAAAGCATA
L. Icterohaemorrhagiae (GU183106) (160) GGATCTGTGATCAACTATTACGGATACGTAAAGCCAGGACAAGCGCCGGACGGTTTAGTCGATGGAAACAAAAAAGCATA
L. Icterohaemorrhagiae (AF245281) (160) GGATCTGTGATCAACTATTACGGATACGTAAAGCCAGGACAAGCGCCGGACGGTTTAGTCGATGGAAACAAAAAAGCATA
L. Bratislava (AY461901)          (158) GGATCTGTGATCAACTATTACGGATACGTAAAGCCAGGACAAGCGCCGGACGGTTTAGTCGATGGAAACAAAAAAGCATA
L. Australis (AY609325)           (160) GGATCTGTGATCAACTATTACGGATACGTAAAGCCAGGACAAGCGCCGGACGGTTTAGTCGATGGAAACAAAAAAGCATA
L. pomona (AY223718)              (145) GGATCTGTGATCAACTATTACGGATACGTAAAGCCAGGACAAGCGCCGGACGGTTTAGTCGATGGAAACAAAAAAGCATA
L. pomona (AY461910)              (158) GGATCTGTGATCAACTATTACGGATACGTAAAGCCAGGACAAGCGCCGGACGGTTTAGTCGATGGAAACAAAAAAGCATA
L. pomona (AY776293)               (67) GGATCTGTGATCAACTATTACGGATACGTAAAGCCAGGACAAGCGCCGGACGGTTTAGTCGATGGAAACAAAAAAGCATA
L. pomona (EU871716)              (160) GGATCTGTGATCAACTATTACGGATACGTAAAGCCAGGACAAGCGCCGGACGGTTTAGTCGATGGAAACAAAAAAGCATA
L. kirschneri (AF121192)          (160) GGATCTGTGATCAACTATTACGGATACGTAAAGCCAGGACAAGCGCCGGACGGTTTAGTCGATGGAAACAAAAAAGCATA
L. Copenhageni (AE016823)         (160) GGATCTGTGATCAACTATTACGGATACGTAAAGCCAGGACAAGCGCCGGACGGTTTAGTCGATGGAAACAAAAAAGCATA
L.muenchen (AHMQ02000025)         (160) GGATCTGTGATCAACTATTACGGATACGTAAAGCCAGGACAAGCGCCGGACGGTTTAGTCGATGGAAACAAAAAAGCATA

                                        240                                                      300
L. canicola (AJ580493)            (240) CTATCTCTATGTTTGGATTCCTGCCGTAATCGCTGAAATGGGAGTTCGTATGATTTCCCCA
L. canicola (DQ092412)            (177) CTATCTCTATGTTTGGATTCCTGCCGTAATCGCTGAAATGGGAGTTCGTATGATTTCCCCA
L. canicola (HM026175)            (240) CTATCTCTATGTTTGGATTCCTGCCGTAATCGCTGAAATGGGAGTTCGTATGATTTCCCCA
L.canicola (AY763509)             (138) CTATCTCTATGTTTGGATCCCAGCTGTAATCGCTGAAATGGGAGTTCGTATGATTTCCCCA
L. canicola (JN831363)            (240) CTATCTCTATGTTTGGATTCCTGCCGTAATCGCTGAAATGGGAGTTCGTATGATTTCCCCA
L. Grippotyphosa (GU592524)       (240) CTATCTCTATGTTTGGATTCCTGCCGTAATCGCTGAAATGGGAGTTCGTATGATTTCCCCA
L. Icterohaemorrhagiae (GU183106) (240) CTATCTCTATGTTTGGATTCCTGCCGTAATCGCTGAAATGGGAGTTCGTATGATTTCCCCA
L. Icterohaemorrhagiae (AF245281) (240) CTATCTCTATGTTTGGATTCCTGCCGTAATCGCTGAAATGGGAGTTCGTATGATTTCCCCA
L. Bratislava (AY461901)          (238) CTATCTCTATGTTTGGATTCCTGCCGTAATCGCTGAAATGGGAGTTCGTATGATTTCCCCA
L. Australis (AY609325)           (240) CTATCTCTATGTTTGGATTCCTGCCGTAATCGCTGAAATGGGAGTTCGTATGATTTCCCCA
L. pomona (AY223718)              (225) CTATCTCTATGTTTGGATTCCTGCCGTAATCGCTGAAATAGGAGTTCGTATGATTTCCCCA
L. pomona (AY461910)              (238) CTATCTCTATGTTTGGATTCCTGCCGTAATCGCTGAAATGGGAGTTCGTATGATTTCCCCA
L. pomona (AY776293)              (147) CTATCTCTATGTTTGGATTCCTGCCGTAATCGCTGAAATGGGAGTTCGTATGATTTCCCCA
L. pomona (EU871716)              (240) CTATCTCTATGTTTGGATTCCTGCCGTAATCGCTGAAATGGGAGTTCGTATGATTTCCCCA
L. kirschneri (AF121192)          (240) CTATCTCTATGTTTGGATTCCTGCCGTAATCGCTGAAATGGGAGTTCGTATGATTTCCCCA
L. Copenhageni (AE016823)         (240) CTATCTCTATGTTTGGATTCCTGCCGTAATCGCTGAAATGGGAGTTCGTATGATTTCCCCA
L.muenchen (AHMQ02000025)         (240) CTATCTCTATGTTTGGATTCCTGCCGTAATCGCTGAAATGGGAGTTCGTATGATTTCCCCA
```

Figure S1: Alignment of Leptospira interrogans sequences from 17 serovars (design adapted from Bourhy P, Bremont S, Zinini F, Giry C, Picardeau M. 2011. Comparison of real-time PCR assays for detection of pathogenic Leptospira spp. in blood and identification of variations in target sequences. J Clin Microbiol 49:2154-60.)
