# Supplementary material for: Quantitative Real-Time PCR Assays for the Detection of Pathogenic Leptospira Species in Urine and Blood Samples in Canine Vaccine Clinical Studies: a Rapid Alternative to Classical Culture Methods
Source: J Clin Microbiol. 2021 Jun 18;59(7):e03006-20. doi: 10.1128/JCM.03006-20 (PMC8218769; doi:10.1128/JCM.03006-20)
Supplement: Supplemental file 2 — Table S1. Download JCM.03006-20-s0002.pdf, PDF file, 69 KB [file jcm.03006-20-s0002.pdf]

Table S1: Specificity of qPCR assay for *Leptospira* for pathogenic and non-pathogenic *Leptospira spp* and reference strains of canine viruses, bacteria and parasites

	Pathogen	***qPCR test result***
Leptospira strains	*L. interrogans* serogroup Canicola	+
	L. interrogans serogroup Grippotyphosa	+
	L. interrogans serogroup Icterohaemorrhagiae	+
	L. interrogans	+
	L. kirschneri serogroup Grippotyphosa	+
	L. fainei	+
	L. biflexa serogroup Patoc	Undetectable
Viral strains	Canine adenovirus type 1	Undetectable
	Canine adenovirus type 2	Undetectable
	Canine parainfluenza virus	Undetectable
	Canine parvovirus	Undetectable
	Feline parvovirus	Undetectable
	Canine distemper virus	Undetectable
Other pathogens	*Bordetella bronchiseptica*	Undetectable
	Leishmania	Undetectable
	Erhlichia canis	Undetectable
	Borrelia burgdoferi	Undetectable
Blood, urine extracts	Negative control	Undetectable

+: presence of an amplification signal; Undetectable: absence of an amplification signal
